# The Prevalence and Incidence of Latent Tuberculosis Infection and Its Associated Factors among Village Doctors in China

**DOI:** 10.1371/journal.pone.0124097

**Published:** 2015-05-21

**Authors:** Guangxue He, Yuan Li, Fei Zhao, Lixia Wang, Shiming Cheng, Hui Guo, John D. Klena, Haiying Fan, Fangfang Gao, Fei Gao, Guoxin Han, Liping Ren, Yudan Song, Yongchao Xiong, Mengjie Geng, Yueyun Hou, Guoming He, Jianbo Li, Shufang Guo, Jun Yang, Daiqin Yan, Yali Wang, Haiyan Gao, Jing An, Xiaoyan Duan, Chunru Wu, Fengming Duan, Dongmei Hu, Kai Lu, Yanlin Zhao, Carol Y. Rao, Yu Wang

**Affiliations:** 1 Chinese Center for Disease Control and Prevention, Beijing, China; 2 Global Disease Detection Branch, Division of Global Health Protection, Center for Global Health, United States Centers for Disease Control and Prevention Atlanta, Georgia, United States of America; 3 Inner Mongolia Center for Tuberculosis Control and Prevention, Hohhot, China; 4 The Tongzhou Maternal & Child Health Hospital, Beijing, China; 5 Ulanqab Center for Tuberculosis Control and Prevention, Jining, China; 6 the Bayannur for Tuberculosis Control and Prevention, Bayannur, China; 7 Linhe District Center for Disease Control and Prevention, Bayannur, China; 8 Hanggin Rear Banner Center for Disease Control and Prevention, Bayannur, China; Institute of Pathogen Biology, CHINA

## Abstract

**Background:**

China is a high tuberculosis (TB) burden country. More than half of acute TB cases first seek medical care in village doctors’ clinics or community health centers. Despite being responsible for patient referral and management, village doctors are not systematically evaluated for TB infection or disease. We assessed prevalence and incidence of latent TB infection (LTBI) among village doctors in China.

**Methods and Findings:**

A longitudinal study was conducted in Inner Mongolia Autonomous Region. We administered a questionnaire on demographics and risk factors for TB exposure and disease; Tuberculin skin testing (TST) and QuantiFERON-TB Gold in-tube assay (QFT-GIT) was conducted at baseline and repeated 12 months later. We used a logistic regression model to calculate adjusted odds ratios (ORs) for risk factors for TST and QFT-GIT prevalence and incidence. At the time of follow up, 19.5% of the 880 participating village doctors had a positive TST and 46.0% had a positive QFT-GIT result. Factors associated with TST prevalence included having a BCG scar (OR = 1.45, 95%*CI* 1.03–2.04) and smoking (OR = 1.69, 95%*CI* 1.17–2.44). Risk factors associated with QFT-GIT prevalence included being male (OR = 2.17, 95%*CI* 1.63–2.89), below college education (OR=1.42, 95%*CI* 1.01–1.97), and working for ≥25 years as a village doctor (OR = 1.64, 95%*CI* 1.12–2.39). The annual incidence of LTBI was 11.4% by TST and 19.1% by QFT-GIT. QFT-GIT conversion was associated with spending 15 minutes or more per patient on average (OR = 2.62, 95%*CI *1.39–4.97) and having BCG scar (OR = 0.53, 95%*CI *0.28–1.00).

**Conclusions:**

Prevalence and incidence of LTBI among Chinese village doctors is high. TB infection control measures should be strengthened among village doctors and at village healthcare settings.

## Introduction

China has the second highest tuberculosis (TB) burden worldwide with a 0.9–1.1 million active TB incidence accounting for 12% of global cases in 2011[1]. Among the new TB cases in China, 5.7% are estimated to have multidrug-resistant TB (MDR-TB); this is as high as 26% among previously treated TB cases[2]. China’s 5th national TB epidemiology survey in 2010 showed more TB cases in rural (active, sputum smear positive, and culture positive pulmonary TB cases were 569/100,000, 78/100,000, and 153/100,000, respectively) than in urban (active, sputum smear positive, and culture positive pulmonary TB cases were 307/100,000, 49/100,000, and 73/100,000, respectively) areas[3]. More than half of rural TB case patients first seek medical care in village doctors’ clinics or township health centers[4]. One report showed that 62.6% of Chinese village doctors had contact with suspected pulmonary TB case patients; contact included registering the cases (75% of village doctors reported this type of contact), referring case-patients to a TB dispensary (77% of village doctors)[5]. After diagnosis and drug regimen prescription, rural TB patients return to village doctors’ clinic to receive the directly observed therapy (DOT) and routine follow up by village doctors[6].

In rural areas of China, village clinics, township health centers, and county hospitals are the foundation of the Chinese healthcare system. Village doctors provide basic medical and public health services (such as vaccinations) to 50.3% of the 1.37 billion Chinese population[7]. In 2011, there were about 1.3 rural healthcare workers (both village doctors and hygienists) per 1000 rural residents[8]. Although village doctors are front-line healthcare providers in rural areas, they are not systematically evaluated for occupational exposures or diseases. Despite their crucial responsibility in TB case referral and management, most Chinese village doctors have no more than a high school education and less than one year of medical training[9]. Their TB control knowledge and awareness is generally poor[10,11]. In addition, rural healthcare facilities do not have adequate personal protective equipment. Data on TB burden associated with Chinese village doctors are urgently needed to facilitate decision-making and resource allocation.

Therefore, we conducted a longitudinal survey to measure prevalence and incidence of latent TB infection (LTBI) among village doctors in Bayan Nur Prefecture of Inner Mongolia Autonomous Region, one of the highest TB burden areas in China[2,3]. In 2011, TB notification from Internet-based National TB Information System indicated that the new sputum smear-positive and active TB case rates were 41/100 000 and 84/100 000, respectively in Bayan Nur Prefecture, higher than the national averages of 28/100 000 and 68/100 000[12].

## Methods

### Study site and population

The longitudinal cohort study was conducted in two counties, Linhe District and Hanggin Rear Banner in Bayan Nur Prefecture, Inner Mongolia Autonomous Region, China. All 880 registered and licensed village doctors from these counties were eligible to participate in the study. A village doctor is defined as a registered and licensed doctor who receives payment for working in a community health center, village clinic or community clinic. The baseline survey was conducted in November 2011 and the follow up survey was administered 12 months later.

On designated days, village doctors were encouraged by public health staff from the local health bureau to visit the study sites. Research assistants interviewed participants using a structured questionnaire, collected basic demographic, occupational and household information, social and medical history, and screened them for clinical symptoms of TB. Participants were examined by chest radiography (CXR) and had blood drawn for Quantiferon (QFT-GIT) testing after which tuberculin was placed for a tuberculin skin test (TST). BCG vaccination status was assessed by visual inspection for a BCG scar. If participant had suspicious symptoms of pulmonary TB or/and CXR was abnormal, then sputum smear/culture tests were implemented. The TB patients were diagnosed based on the clinical symptoms and related exams.

### TST and QFT-GIT procedures

We performed a single-step TST using 0.1 ml [5 international units (IU)] tuberculin, (Chengdu Institute of Biological Products, Chengdu, China). TST was administered using the Mantoux method; skin reactions were read 72 h after TST placement. We considered TST induration size > 10mm as positive[13]. The presence of a BCG scar was verified by a nurse. All TST-positive village doctors were advised to have close follow up for development of any active TB symptoms; prophylaxis treatment for latent TB infection is not routinely performed in China.

QFT-GIT testing was performed and interpreted according to the manufacturer’s instructions (Qiagen, Valencia, CA, USA). Blood samples were collected, incubated at 37°C for 24 h (within 16 h of collection), centrifuged and stored at 4°C locally for up to 14 days. The samples were transported every two weeks to the testing laboratory in Ulanqab in Inner Mongolia, where the enzyme-linked immune sorbent assay (ELISA) was performed manually in batches. The interferon-gamma (IFN-γ) response level, measured in IU/ml, was determined by measuring the amount of IFN-γ elaborated in response to the TB antigens ESAT-6, CFP-10 and TB7.7 (TB antigen) minus the amount present in the negative control (Nil); a positive result was defined as TB antigen-Nil >0.35 IU/ml and >25% of the negative control. Results were indeterminate if the response to the positive control (mitogen-Nil) was <0.5 IU/ml or the negative control (Nil) was >8 IU/ml[14]. Results were interpreted as negative if these criteria were not met. Samples with indeterminate results were tested a second time, and the results of the second test were recorded as the final result. As the QFT-GIT ELISA cannot determine absolute values of IFN-γ ≥ 10 IU/ml, these results were recorded as >10 IU/ml. Laboratory quality and procedures were overseen by an external laboratory expert trained by the manufacturer.

In order to implement the survey successfully, project coordination and technical groups were established. The coordination group was responsible for organizing and coordinating the implementation of the study. The technical group was responsible for the training of project staffs, quality control, and checking. A research group was established in each study site and was responsible for organizing the field work and for quality control. The research group included TB control staff, TST and QFT-GIT technicians and a deputy director of a TB center as the head of the group. TST and QFT-GIT testing were performanced skillfully by specified technicians who had were trained by international experts since 2009. We used three criteria for determining conversion of previously LTBI-negative cases by TST and four criteria for conversion to LTBI-by QFT-GIT. We defined TST conversion as: 1) baseline TST <10mm and follow up TST >10mm; 2) baseline TST <10mm and follow up TST >10mm and a 6mm increase over the baseline; 3) baseline TST <10mm and follow up TST >10mm and a 10mm increase over the baseline[15]. We defined QFT-GIT conversion as: 1) baseline TB antigen-Nil <0.35 IU/ml and follow up TB antigen-Nil ≥0.35 IU/ml, without any consideration of the magnitude in change of the TB antigen-Nil response; 2) baseline TB antigen-Nil <0.35IU/ml and follow up TB antigen-Nil ≥0.35IU/ml, plus a 30% increase over the baseline; 3) baseline TB antigen-Nil <0.35IU/ml and follow up TB antigen-Nil ≥0.35IU/ml, plus an absolute increase of 0.35IU/ml over the baseline; 4) baseline TB antigen-Nil<0.35IU/ml and follow up TB antigen-Nil ≥0.70IU/ml[15]. We defined QFT-GIT reversion as having a baseline TB antigen-Nil ≥0.35 IU/ml and a follow up TB antigen-Nil level <0.35 IU/ml.

### Ethics approval

The research protocol was approved by the Chinese Ethical Committee for Tuberculosis Operational Research and the Chinese Center for Disease Control and Prevention. Approval was also obtained from CDC per CDC policies and procedures and relevant regulations[16]. Written informed consent was obtained of each village doctor prior to participating in the survey.

### Statistical analysis

Data were double entered using EpiData3.1 and checked for errors. Statistical analyses were performed using SAS, version 9.1 (SAS Institute, Cary, NC, USA). We dichotomized TST and QFT-GIT results into positive and negative; indeterminate QFT-GIT results were excluded from the comparison. We assessed the level of agreement between QFT-GIT results and TST induration cut-offs using Cohen’s kappa coefficient (k). k>0.75 is generally considered as good consistency, and k<0.4 indicates poor agreement.

We used bivariate analysis to calculate unadjusted odds ratios (ORs) and 95% confidence intervals (CIs) for characteristics associated with prevalence of LTBI (i.e., positive TST or QFT-GIT) and annual incidence of LTBI (i.e., TST or QFT-GIT conversion). Risk factors associated with infection as indicated by the literature and additional factors with p-value < 0.20 in bivariate analysis on TST or QFT-GIT results using backward stepwise multivariable logistic regression.

## Results

### Prevalence of LTBI and associated risk factors

At the time of follow up, all 880 registered village doctors in the two counties completed questionnaires; 876 completed QFT-GIT testing, 875 TST and 875 completed both tests. The median age was 40 years (range 19, 77); 459 (52.2%) were men, and 653(74.2%) had less than college education. The average duration of time as a village doctor was 18 years (range 0.5–56.3). Six hundred and twenty-nine (71.5%) participants had an average yearly income over 20,000 RMB (about 3300 USD). Twenty (2.3%) village doctors reported having a previous diagnosis of TB, including 13 cases that were reported in 2011; 320 (36.4%) had BCG scar (Table 1).

**Table 1:** Characteristics of 880 village doctors in 2012.

Characteristic	Participants n, (%)
**Country**	
Hanggin Rear Banner	428 (48.6)
Linhe District	452 (51.4)
**Age, years**	
17–29	88 (10.0)
30–39	350 (39.8)
40–49	282 (32.0)
>50	160 (18.2)
**Gender**	
Female	421 (47.8)
Male	459 (52.2)
**Education**	
College or above	227 (25.8)
Below college	653 (74.2)
**Working duration, years**	
<15	380 (43.2)
15~	300 (34.1)
25~	200 (22.7)
**Average yearly income**	
<20,000 RMB*	251 (28.5)
>20,000 RMB	629 (71.5)
**Smoke**	
No	667 (75.8)
Yes	213 (24.2)
**Previously diagnosed with TB disease**	
No	860 (97.7)
Yes	20 (2.3)
**BCG scar**	
No	560 (63.6)
Yes	320 (36.4)

*20,000 RMB is about 3300 USD

At the time of follow up, 19.5% of the village doctors (171/875) had positive TST results (cut-off value of ≥ 10 mm). By bivariate analysis, associated factors with positive TST results included being male, having a visible BCG scar and current smoking. In multivariate analysis, factors associated with TST positivity included having a visible BCG scar (OR = 1.45, 95%*CI* 1.03–2.04) and current smoking (OR = 1.69, 95% *CI* 1.17–2.44) (Table 2).

**Table 2:** The prevalence of LTBI detected by TST (>10mm) and its associated factors among village doctors in 2012.

	n/N(%)	OR(95% *CI*)	*P*	aOR(95% *CI*)
**Gender**				
Female	68/419(16.2%)	Referent		
Male	103/456(22.6%)	1.51(1.07–2.12)	0.018	
**Age, years**				
<40	88/435(20.2%)	Referent		
>40	83/440(18.9%)	0.92(0.66–1.28)	0.610	
**County**				
Hanggin Rear Banner	80/423(18.9%)	Referent		
Linhe District	91/452(20.1%)	1.08(0.77–1.51)	0.649	
**Education**				
College or above	42/225(18.7%)	Referent		
Below college	129/650(19.8%)	1.08(0.73–1.59)	0.701	
**Working duration, years**				
<15	80/377(21.2%)	Referent		
15~	49/298(16.4%)	0.73(0.49–1.08)	0.118	
25~	42/200(21.0%)	0.99(0.65–1.50)	0.951	
**Minutes spend on diagnosing one patient, minutes**				
<15	84/473(17.8%)	Referent		
>15	87/402(21.6%)	1.28(0.92–1.79)	0.149	
**Whether referred suspected TB patient in recent three years**				
No	134/681(19.7%)	Referent		
Yes	37/194(19.1%)	0.96(0.64–1.44)	0.851	
**Density of clinical areas(m** ^**2**^ **per staff)**				
<18	92/445(20.7%)	Referent		
>18	79/430(18.4%)	0.86(0.62–1.21)	0.391	
**Average yearly income(10,000 RMB)**				
<2	44/248(17.7%)	Referent		
>2	127/627(20.3%)	1.18(0.81–1.72)	0.398	
**Density of household (person number per room)**				
<1	104/482(21.6%)	Referent		
1	42/233(18.0%)	0.80(0.54–1.19)	0.270	
>1	25/160(15.6%)	0.67(0.42–1.09)	0.105	
**BCG scar**				
No	97/555(17.5%)	Referent		Referent
Yes	74/320(23.1%)	1.42(1.01–2.00)	0.043	1.45(1.03–2.04)
**Smoking status**				
No	116/664(17.5%)	Referent		Referent
Yes	55/211(26.1%)	1.67(1.15–2.40)	0.006	1.69(1.17–2.44)

At the time of follow up, among the 876 who completed QFT-GIT, ten subjects with indeterminate results were excluded. Forty-six percent of village doctors tested (398/866) had positive QFT-GIT results. By bivariate analysis, associated factors with a positive QFT-GIT included being male, age ≥40 years, living in Linhe district, having worked as a village doctor for ≥15 years, spending 15 minutes or more on diagnosing a patient, and current smoking (Table 3). In multivariate analysis, using 2012 data, risk factors associated with QFT-GIT positivity included being male (OR = 2.17, 95%*CI* 1.63–2.89), living in Linhe district (OR = 2.69, 95%*CI* 2.02–3.58), having less than a college education (OR = 1.42, 95%*CI* 1.01–1.97), working for ≥25 years as a village doctor (OR = 1.64, 95%*CI* 1.12–2.39) (Table 3).

**Table 3:** The prevalence of LTBI detected by QFT-GIT and its associated factors among village doctors in 2012.

	n/N(%)	OR(95% *CI*)	*P*	aOR(95% *CI*)
**Gender**				
Female	148/415(35.7%)	Referent		Referent
Male	250/451(55.4%)	2.24(1.71–2.95)	0.000	2.17(1.63–2.89)
**Age, years**				
<40	175/432(40.5%)	Referent		
>40	223/434(51.4%)	1.55(1.19–2.03)	0.001	
**County**				
Hanggin Rear Banner	146/423(34.5%)	Referent		Referent
Linhe District	252/443(56.9%)	2.50(1.90–3.30)	0.000	2.69(2.02–3.58)
**Education**				
College or above	93/225(41.3%)	Referent		Referent
Below college	305/641(47.6%)	1.29(0.95–1.75)	0.106	1.42(1.01–1.97)
**Working duration, years**				
<15	148/374(39.6%)	Referent		Referent
15~	140/295(47.5%)	1.38(1.01–1.88)	0.041	1.23(0.89–1.71)
25~	110/197(55.8%)	1.93(1.36–2.74)	0.000	1.64(1.12–2.39)
**Minutes spend on diagnosing one patient, minutes**				
<15	182/470(38.7%)	Referent		
>15	216/396(54.5%)	1.90(1.45–2.49)	0.000	
**Whether referred suspected TB patient in recent three years**				
No	297/672(44.2%)	Referent		
Yes	101/194(52.1%)	1.37(1.00–1.89)	0.053	
**Density of clinical areas(m** ^**2**^ **per staff)**				
<18	239/437(54.7%)	Referent		
>18	159/429(37.1%)	0.49(0.37–0.64)	0.000	
**Average yearly income(10,000 RMB)**				
<2	116/243(47.7%)	Referent		
>2	282/623(45.3%)	0.91(0.67–1.22)	0.512	
**Density of household (person number per room)**				
<1	221/478(46.2%)	Referent		
1	108/229(47.2%)	1.04(0.76–1.42)	0.817	
>1	69/159(43.4%)	0.89(0.62–1.28)	0.534	
**Visible BCG scar**				
No	278/546(50.9%)	Referent		
Yes	120/320(37.5%)	0.58(0.44–0.77)	0.000	
**Smoking status**				
No	288/659(43.7%)	Referent		
Yes	110/207(53.1%)	1.46(1.07–2.00)	0.018	

### Incidence of LTBI and its risk factors

**Fig 1:**
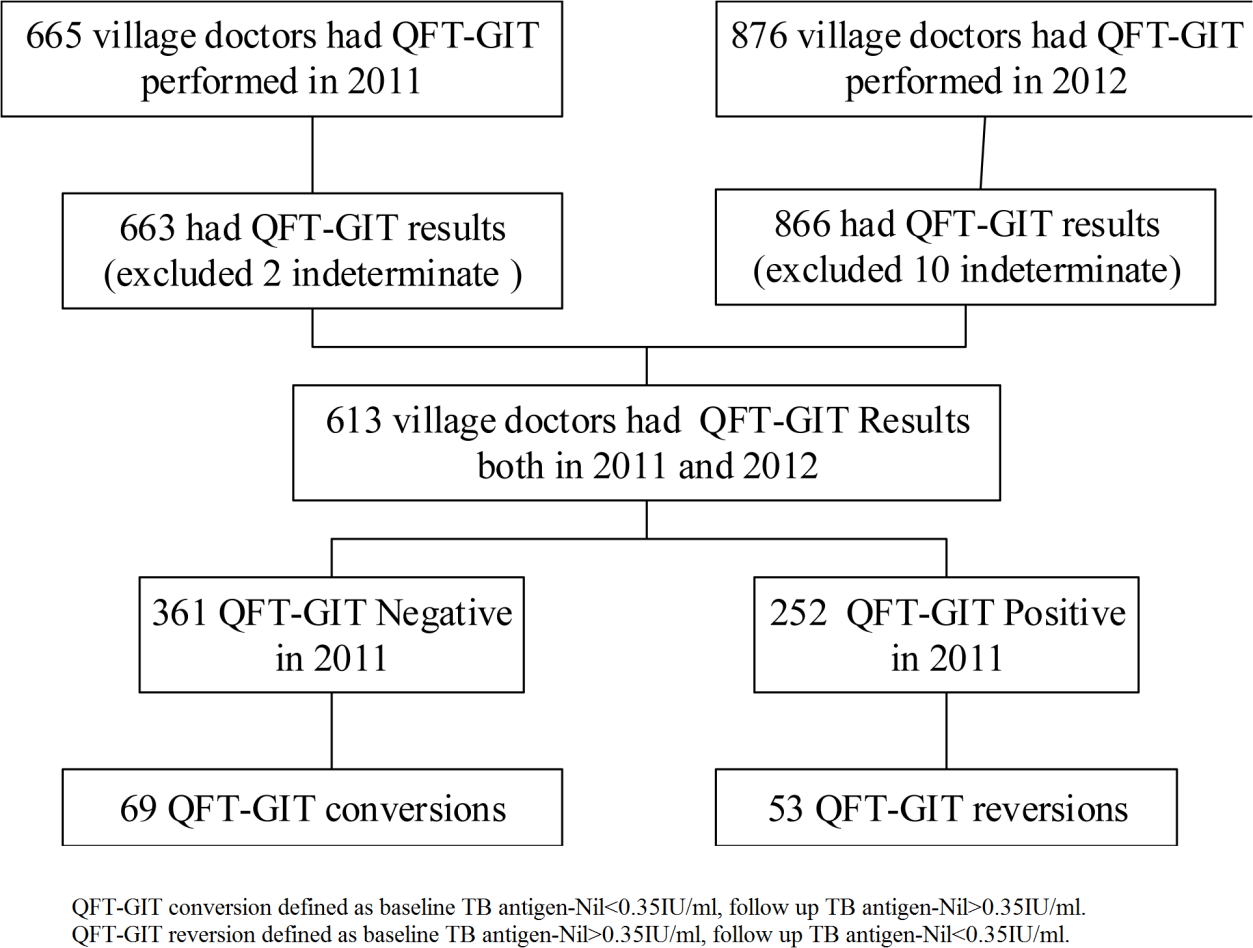
The QFT-GIT results of a baseline cross-sectional survey in December 2011 and the follow-up survey in December 2012 of village doctors in two counties in the Inner Mongolia Autonomous Region, China.

Of the 875 village doctors who completed TST at follow up, 618 had a baseline TST result. Of the 866 village doctors who had QFT-GIT results at follow up, 619 had a baseline QFT-GIT performed and one with an indeterminate QFT-GIT result was excluded at baseline. For baseline TST results, 75.2% (465/618) were negative (had TST induration size < 10 mm). For baseline QFT-GIT results, 58.4% (361/618) had negative results. A total of 613 participants had QFT-GIT results both at baseline (in 2011) and at follow-up (in 2012) (Fig 1).

Based on TST results, LTBI incidence for the 465 previously negative village doctors ranged from 8.0% to 11.4%, depending on how conversion by TST was defined (Table 4). In multivariate analysis, the TST conversion (using conversion rate of 11.4%) was associated with a visible BCG scar (OR = 1.82, 95% *CI* 1.00–3.33), while working between 15 years to 25 years was protective (OR = 0.46, 95% *CI* 0.22–0.96) (Table 5).

**Table 4:** The incidence of LTBI detected by TST and QFT-GIT among village doctors.

Method	Criteria for conversion	Number Negative in 2011(N)	Number of Conversions (n)	Annual incidence of LTBI(n/N)	Conversion rate(100 person-years)
TST	Baseline:TST<10mm, Follow up:TST>10mm	465	53	11.4	11.4
	Baseline: TST<10mm, Follow up: TST>10mm, plus a 6mm increase over baseline	465	45	9.7	9.7
	Baseline: TST<10mm, Follow up: TST>10mm, plus a 10mm increase over baseline	465	37	8.0	8.0
QFT-GIT	Baseline: TB antigen-Nil<0.35IU/ml, Follow up: TB antigen-Nil>0.35IU/ml	361	69	19.1	19.1
	Baseline: TB antigen-Nil<0.35IU/ml, Follow up: TB antigen-Nil>0.35IU/ml,plus a 30% increase over baseline	361	68	18.8	18.8
	Baseline: TB antigen-Nil<0.35IU/ml, Follow up: TB antigen-Nil>0.35IU/ml, plus an absolute increase of 0.35IU/ml over baseline	361	63	17.5	17.5
	Baseline: TB antigen-Nil<0.35IU/ml, Follow up: TB antigen-Nil>0.70IU/ml	361	52	14.4	14.4

**Table 5:** Factors associated with LTBI conversion detected by TST (n = 465)^*^.

	n/N(%)	OR(95% *CI*)	*P*	aOR(95% *CI*)
**Gender**				
Female	22/236(9.3%)	Referent		
Male	31/229(13.5%)	1.52(0.85–2.72)	0.155	
**Age, years**				
<40	30/222(13.5%)	Referent		
>40	23/243(9.5%)	0.67(0.38–1.19)	0.172	
**County**				
Hanggin Rear Banner	43/347(12.4%)	Referent		
Linhe District	10/118(8.5%)	0.66(0.32–1.35)	0.250	
**Education**				
College or above	11/89(12.4%)	Referent		
Below college	42/376(11.2%)	0.89(0.44–1.81)	0.751	
**Working duration, years**				
<15	28/197(14.2%)	Referent		Referent
15~	11/158(7.0%)	0.45(0.22–0.94)	0.033	0.46(0.22–0.96)
25~	14/110(12.7%)	0.88(0.44–1.75)	0.717	1.07(0.52–2.20)
**Minutes spend on diagnosing one patient, minutes**				
<15	35/312(11.2%)	Referent		
>15	18/153(11.8%)	1.06(0.58–1.93)	0.862	
**Whether referred suspected TB patient in recent three years**				
No	43/359(12.0%)	Referent		
Yes	10/106(9.4%)	0.77(0.37–1.58)	0.470	
**Density of clinical areas(m** ^**2**^ **per staff)**				
<18	13/135(9.6%)	Referent		
>18	40/330(12.1%)	1.29(0.67–2.51)	0.444	
**Average yearly income(10,000 RMB)**				
<2	9/106(8.5%)	Referent		
>2	44/359(12.3%)	1.51(0.71–3.19)	0.286	
**Density of household (person number per room)**				
<1	35/268(13.1%)	Referent		
1	13/115(11.3%)	0.85(0.43–1.67)	0.635	
>1	5/82(6.1%)	0.43(0.16–1.14)	0.091	
**Visible BCG scar**				
No	24/265(9.1%)	Referent		Referent
Yes	29/200(14.5%)	1.70(0.96–3.03)	0.070	1.82(1.00–3.33)
**Smoking status**				
No	38/359(10.6%)	Referent		
Yes	15/106(14.2%)	1.39(0.73–2.64)	0.312	

*The criterion used for incidence was baseline TST<10mm, follow up TST>10mm.

Based on QFT-GIT results, LTBI incidence for the 361 previously negative village doctors ranged from 14.4% to 19.1%, depending upon how conversion by QFT-GIT was defined (Table 4). By bivariate analysis, risk factors associated with QFT-GIT defined conversion (19.1%) included being male, living in Linhe district, spending 15 minutes or more on diagnosing a patient, crowded clinical areas (<18 m^2^/staff), smoking and no BCG scar. In multivariate analysis, risk factors associated with QFT-GIT positive conversion were living in Linhe district (OR = 6.44, 95% *CI* 3.33–12.43), spending 15 minutes or more on diagnosing a patient (OR = 2.62, 95% *CI* 1.39–4.97), while having a visible BCG scar was protective (OR = 0.53, 95% *CI* 0.28–1.00) (Table 6).

**Table 6:** Factors associated with LTBI conversion detected by QFT-GIT (n = 361)^*^.

	n/N(%)	OR(95% *CI*)	*P*	aOR(95% *CI*)
**Gender**				
Female	29/197(14.7%)	Referent		
Male	40/164(24.4%)	1.87(1.10–3.18)	0.021	
**Age, years**				
<40	41/208(19.7%)	Referent		
>40	28/153(18.3%)	0.91(0.54–1.56)	0.736	
**County**				
Hanggin Rear Banner	19/253(7.5%)	Referent		Referent
Linhe District	50/108(46.3%)	10.62(5.82–19.37)	0.000	6.44(3.33–12.43)
**Education**				
College or above	18/88(20.5%)	Referent		
Below college	51/273(18.7%)	0.89(0.49–1.63)	0.713	
**Working duration, years**				
<15	37/185(20.0%)	Referent		
15~	19/121(15.7%)	0.75(0.41–1.37)	0.343	
25~	13/55(23.6%)	1.24(0.60–2.54)	0.560	
**Minutes spend on diagnosing one patient, minutes**				
<15	26/248(10.5%)	Referent		Referent
>15	43/113(38.1%)	5.25(3.01–9.15)	0.000	2.62(1.39–4.97)
**Whether referred suspected TB patient in recent three years**				
No	53/294(18.0%)	Referent		
Yes	16/67(23.9%)	1.43(0.76–2.69)	0.273	
**Density of clinical areas(m** ^**2**^ **per staff)**				
<18	45/115(39.1%)	Referent		
>18	24/246(9.8%)	0.17(0.10–0.30)	0.000	
**Average yearly income(10,000 RMB)**				
<2	21/86(24.4%)	Referent		
>2	48/275(17.5%)	0.65(0.37–1.17)	0.154	
**Density of household (person number per room)**				
<1	34/202(16.8%)	Referent		
1	22/90(24.4%)	1.60(0.87–2.93)	0.129	
>1	13/69(18.8%)	1.15(0.57–2.33)	0.704	
**Visible BCG scar**				
No	50/193(25.9%)	Referent		Referent
Yes	19/168(11.3%)	0.37(0.21–0.65)	0.001	0.53(0.28–1.0)
**Smoking status**				
No	47/281(16.7%)	Referent		
Yes	22/80(27.5%)	1.89(1.06–3.38)	0.032	

* The criterion used for incidence was baseline TB antigen-Nil<0.35IU/ml, follow up TB antigen-Nil>0.35IU/ml

Among the 252 participants with QFT-GIT positive baseline results (>0.35 IU/ml), 53 (21.0%) appeared to revert at the time of follow up (Fig 1).

### Agreement between TST and QFT-GIT results

At follow up, 865 participants of the 880 village doctors finished both TST and QFT-GIT testing with effective results. The agreement between these test results was 62.9%, with a Kappa value = 0.220 (95% *CI* 0.17–0.28), indicating poor consistency (Table 7).

**Table 7:** Agreement between TST and QFT-GIT Results in 2012.

TST>10mm	QFT-GIT		Total
	Negative	Positive	
Negative	422(48.8%)	276(31.9%)	698(80.7%)
Positive	45(5.2%)	122(14.1%)	167(19.3%)
Total	467(5.2%)	398(14.1%)	865(100.0%)

Kappa value = 0.220 (95%CI (0.165–0.275))

## Discussion

This is first systematic study using QFT-GIT to assess the prevalence and incidence of LTBI among Chinese village doctors. Prevalence of LTBI detected by QFT-GIT was 46.0% among village doctors; this is higher than the TB infection rate in the general population, detected by TST, during a nationwide tuberculosis epidemiology survey conducted in 2000[4]. Moreover the incidence of LTBI detected by QFT-GIT ranged between 14.4–19.1% among village doctors, indicating ongoing transmission in village doctor healthcare settings. This is much higher than the incidence of LTBI (1.34%) estimated based on the year 2000 nationwide tuberculosis epidemiology survey and other studies[17–19]. Sputum smear positive patients continue to be a source of infection in villages; estimates are that an infectious patient will infect 10 to 15 individuals each without proper treatment[20]. Given that most people living in rural China consult village doctors as their first point of contact regarding the detection, treatment and monitoring of TB, the risk of transmission of TB from patients to village doctors is likely to remain high.

Work-related exposures, such as ≥15 years working experience as a village doctor, relatively long contact exposures with a patient (15 minutes or more to make a diagnosis) and being a male village doctor were associated with higher prevalence of LTBI; these risk factors are consistent with a number of international studies[17,18]. A previous study found that LTBI prevalence was 69% among traditional HCWs in two hospitals in Inner Mongolia, and infection was also associated with work-related exposures[21]. We also found current smoking, an urban working location (Linhe district) and crowded working conditions were associated with higher risk of LTBI. Current smoking has been shown to be a risk factor for TB in previous studies[21,22]. Other studies reported that even low-cost strategies to reduce TB transmission are rarely implemented in health care facilities in low-income countries[17,19]. It is essential that routine surveillance among all HCWs, including village doctors, be conducted in China, and reduction of the risk of transmission must be given high priority as occupational TB has the potential to lead to loss of essential, skilled village doctors.

BCG vaccination was shown to be a protective factor in preventing LTBI detection by QFT-GIT in this study. The possibility that BCG vaccination can prevent acquisition of TB infection has previously been reported in a study that evaluated children exposed to TB using an IGRA[23]. Childhood BCG vaccination coverage in China now approaches 100%; therefore, future generations of village doctors may benefit, if such a protective effect is consistently demonstrated.

We found that the LTBI rates detected by QFT-GIT are higher than that by TST. Surveys of health care workers (HCWs) using IGRA in other high TB burden countries such as India, Russia and Viet Nam, have also found high TB infection rates, ranging from 40% to 47%; one survey in the Republic of Georgia found a prevalence of 60%[24–27]. Surveys of HCWs in low- and middle-income countries utilizing internationally standardized tuberculin antigen preparations have found LTBI prevalence ranging from 33% to 79%[17]. Comparison of IGRA and TST results in some high-burden countries in general has shown that both methods provide similar prevalence estimates. However, the use of IGRA among HCWs is limited by high conversion and reversion rates; these are important considerations when performing serial testing, as is often done with HCWs[18].

There are several limitations to our study. There are no reports preceding this study on LTBI rates specifically among village doctors; LTBI rates are only known among HCWs in hospitals and in the general population, making direct comparison of the LTBI rates between village doctors challenging. The tuberculin antigen used for the study was produced in China and is not internationally standardized making comparisons to other studies using standardized tuberculin antigen difficult and this may have adversely affected the agreement between TST and QFT-GIT. There is no consensus in the literature on interpreting serial QFT-GIT assays[28]; this has resulted in our using a range of values for the QFT-GIT positive percentages. Finally no gold standard tool for detecting LTBI currently exists; development and validation of new tools that have the promise of high sensitivity and specificity to detect LTBI are critical.

## Conclusion

This survey of LTBI among Chinese village doctors used both TST and QFT-GIT. We found that the prevalence and incidence of LTBI among village doctors is high; we found that several work-related risk factors such as working location and duration, and length of exposure to potentially infected patients were significant risk factors. TB infection control interventions should be implemented and strengthened among village doctors and in their clinics. Training targeting TB infection control may help reduce transmission of TB in peripheral healthcare settings.

## Supporting Information

S1 Table Ethics approval in Chinese in 2010. (PDF)

S2 Table Ethics approval in Chinese in 2011. (PDF)
