# Supplementary material for: The Prevalence and Incidence of Latent Tuberculosis Infection and Its Associated Factors among Village Doctors in China
Source: PLoS One. 2015 May 21;10(5):e0124097. doi: 10.1371/journal.pone.0124097 (PMC4440671; doi:10.1371/journal.pone.0124097)
Supplement: S1 Table — (PDF) [file pone.0124097.s001.pdf]

伦理委员会
研究方案审核表

编号:

研究题目	中美结核感染控制合作项目—对2009-2010内蒙古结核病项目进行随访追踪		
研究单位	中国疾病预防控制中心·结核病预防控制中心·	任务来源	中美新发和再发传染病合作项目
所属专业	结核病学	课题负责人	王黎霞
审查方式	☐书面审查; ☑会议审查;	日　期	2011.8.12
主任委员	万利亚	副主任委员	何广学
评审委员	成诗明.端木宏谨.赵雁林.宋文质.邵仁宗.周林		
申请书的批件及编号			
目前已获得的研究相关资料	项目实施方案、知情同意书.和调查问卷		

课题负责人资格评价: 符合条件
设计方案评价: 设计合理
知情同意书及措施评价: 符合要求
提供医疗措施评价: 措施合理
受试者补偿/赔偿措施评价: 有补偿措施
利益冲突评价: 无

委员名称	评审意见（同意、修改后再审或不同意）	签名	日期
万利亚	同意.	万利亚	2011.8.12
端木宏谨	同意	[illegible]	2011.8.12
赵雁林	同意	[illegible]	2011.8.12
邵仁宗	同意	[illegible]	2011.8.12
周林	同意	[illegible]	2011.8.12

宋文质	北京大学医学院公共卫生学院	宋文质	2011.8.12
	同意		
[illegible]	同意	[illegible]	2011.8.12
何[illegible]学	同意	[illegible]	2011.8.12

结论	评审意见
1、批准	同意.
2、作必要修改后再审	
3、不批准	
主任委员签字:	万利亚
副主任委员签字:	何[illegible]学
记录人签字:	侯月云
日期	2011.8.12

盖章: 伦理委员会

日期:
