# Supplementary material for: The Prevalence and Incidence of Latent Tuberculosis Infection and Its Associated Factors among Village Doctors in China
Source: PLoS One. 2015 May 21;10(5):e0124097. doi: 10.1371/journal.pone.0124097 (PMC4440671; doi:10.1371/journal.pone.0124097)
Supplement: S2 Table — (PDF) [file pone.0124097.s002.pdf]

伦理委员会
研究方案审核表

编号:

研究题目	中美结核感染控制合作项目—继续加强医务人员结核感染控制调查		
研究单位	中国疾病预防控制中心结核病预防控制中心	任务来源	中美新发和再发传染病合作项目
所属专业	结核病学	课题负责人	王黎霞
审查方式	☐书面审查; ☑会议审查;	日 期	2011.8.12
主任委员	万利亚	副主任委员	何广学
评审委员	成诗明、端木宏谨、赵雁林、宋媛、邱宗、周林		
申请书的批件及编号			
目前已获得的研究相关资料	项目实施方案、知情同意书和调查问卷		

课题负责人资格评价: 符合条件

设计方案评价: 设计合理

知情同意书及措施评价: 符合要求

提供医疗措施评价: 措施合理

受试者补偿/赔偿措施评价: 有补偿措施

利益冲突评价: 无

委员名称	评审意见(同意、修改后再审或不同意)	签名	日期
万利亚	同意	万利亚	2011.8.12
端木宏谨	同意	端木宏谨	2011.8.12
赵雁林	同意	赵雁林	2011.8.12
邱宗	同意	邱宗	2011.8.12
周林	同意	周林	2011.8.12

[illegible]	北京大学医学部公共卫生学院 同意	[illegible]	2011.8.12
[illegible]	中国CDC 同意	[illegible]	2011.8.12
[illegible]	中国CDC 同意	[illegible]	2011.8.12

结论	评审意见
1、批准	同意。
2、作必要修改后再审	
3、不批准	
主任委员签字:	[illegible]
副主任委员签字:	[illegible]
记录人签字:	[illegible]
日期	2011.8.12

盖章：伦理委员会

日期:
